# An In Vivo Study of LNS8801, a GPER Agonist, in a Spontaneous Melanoma‐Prone Mouse Model, TGS

**DOI:** 10.1111/pcmr.13197

**Published:** 2024-09-16

**Authors:** Christina Marinaro, John Sauer, Christopher A. Natale, Todd Ridky, Suzie Chen

**Affiliations:** ^1^ Susan Lehman Cullman Laboratory for Cancer Research, Ernest Mario School of Pharmacy Rutgers University, The State University of New Jersey New Brunswick New Jersey USA; ^2^ U.S. Department of Veterans Affairs East Orange New Jersey USA; ^3^ Rowan‐Virtua School of Osteopathic Medicine Rowan University Stratford New Jersey USA; ^4^ Linnaeus Therapeutics Inc. Haddonfield New Jersey USA; ^5^ Department of Dermatology, Perelman School of Medicine University of Pennsylvania Philadelphia Pennsylvania USA; ^6^ Rutgers Cancer Institute of New Jersey New Brunswick New Jersey USA

**Keywords:** estrogen receptor, Grm1, LNS8801, melanocytes, melanoma, mouse model, transgenic

## Abstract

Melanoma is the most aggressive and deadly form of skin cancer that arises from the transformation of melanocytes, the pigment producing cells of the skin. In the year 2024 there will be approximately 10,000 new cases of melanoma diagnosed and approximately 8,000 deaths attributed to melanoma in the United States. In this study we treated a group of male and female transgenic mice that spontaneously develop metastatic melanoma, TGS, with a G‐protein‐coupled estrogen receptor agonist LNS8801 to assess the efficacy on disease progression. A second group of male and female TGS mice was also exposed to UVB irradiation to mimic exposure to sunlight. Over the course of the 32‐week experiment, visible images were taken by the small animal imaging IVIS system to track tumor progression, and blood and tissue samples were collected for molecular analyses. Results showed that sex‐biased effects were observed in the efficacy of LNS8801 and that LNS8801 shows a UV‐protective influence in both male and female TGS mice.

## Graphical abstract

TGS mice when exposed to UV radiation and treated with LNS8801 have a significantly lower tumor burden than those exposed to UV and treated with the vehicle.

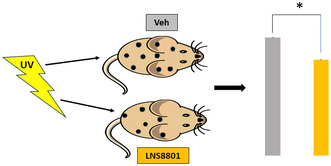

**Summary**

- Experimental animal models have been the gold standard to evaluate the efficacy of therapeutic compounds for human diseases; however, there are very few models with intact immune systems for longitudinal studies.
- For this study, we used a melanoma‐prone, immunocompetent mouse model to assess the consequences of treatment with an agonist of G‐protein‐coupled estrogen receptor.
- The results highlight the need to compare short‐term versus long‐term in vivo studies with different treatment options for melanoma and other cancers.
- The goal of this research is to translate to the clinic to provide treatment with enhanced efficacious outcomes, minimum toxicity and to increase the quality of life for melanoma patients.

## 1 Introduction

Our group previously described an abnormally expressed normal neuronal receptor, metabotropic glutamate receptor 1 (protein‐mGluR1/gene‐Grm1), in melanocytes that led to cell transformation *in vitro* and metastatic tumor formation *in vivo* (Pollock et al. 2003). Grm1 is a G‐Protein‐Coupled Receptor (GPCR) that is normally expressed and functions in the central nervous system (Aiba et al. 1994; Ménard and Quirion 2012). When activated by its natural ligand, L‐glutamate, the receptor undergoes a conformational change, exchanging GTP for GDP that activates phospholipase C (PLC). PLC hydrolyzes phosphatidylinositol (4.5)‐biphosphate (PIP2) to diacylglycerol (DAG) and inositol 1,4,5‐triphosphate (IP3), which act as secondary messengers and trigger the release of calcium into the cytosol and activates protein kinase C (PKC) (Lüscher and Huber 2010; Taylor et al. 1991). Activated PKC initiates a phosphorylation cascade that stimulates the mitogen‐activated protein kinase (MAPK) and phosphatidylinositide 3‐kinase/AKT (PI3K/AKT) pathways, leading to cell proliferation and cell survival (Schönwasser et al. 1998).

Estrogen has been postulated to play a role in the prevention of the transformation of melanocytes to melanoma. Activation of the G‐Protein‐Coupled Estrogen Receptor (GPER), a GPCR, on melanocytes is predicted to be the mediator of this process (Natale et al. 2018; Revankar et al. 2005). In melanocytes, estrogen signaling is mediated mostly through GPER which is distinct from the classical estrogen pathways (Filardo et al. 2002). Increased estrogen levels in melanocytes enhance pigmentation, and differentiation and decrease cell proliferation (Natale et al. 2018). Melanocortin receptor 1 (MC1R) is a GPCR that regulates both pigmentation and differentiation in melanocytes. MC1R is stimulated upon binding to a class of pituitary peptide hormones (melanocortins) including melanocyte‐stimulating hormone and adrenocorticotropic hormone, which then activates adenylyl cyclase and upregulates the production of cyclic adenosine monophosphate (cAMP) (Abdel‐Malek et al. 1999). Elevated levels of cAMP initiate a cascade of activities via activation of protein kinase A (PKA), this then triggers cAMP response element‐binding protein (CREB), a transcription factor that promotes the transcription of microphthalmia‐associated transcription factor (MITF), the master regulator of genes required for melanin synthesis (D'Orazio and Fisher 2011). Suggesting that GPER is a valid target to upregulate melanocyte differentiation and down‐regulate melanoma tumor progression.

G‐1, a nonsteroid, is a very selective high‐affinity agonist of GPER (Bologa et al. 2006). G‐1 only activates GPER, not classical estrogen receptors. G‐1 has been used to treat multiple models of cancer including cervical, lung, and prostate cancer (Chan et al. 2010; Kurt, Çelik, and Kelleci 2015; Zhang et al. 2015). When GPER is activated by G‐1, there is an increase in the cytokine IL‐10 production mediated through the MAPK/ERK pathway (Brunsing and Prossnitz 2011). Earlier studies demonstrated that in the estrogen receptor (ER)‐positive (MCF‐7) and ER‐negative (SKBr3) breast cancer cell lines, inclusion of G‐1 in the growth media inhibited cell proliferation and induced cell cycle arrest in the G2/M phase (Ribeiro, Santos, and Custódio 2017; Weißenborn et al. 2014). Ridky and co‐workers demonstrated that systemically delivered G‐1 was well‐tolerated in melanoma xenografts and allografts and also extended the survival of tumor‐bearing mice. Not only is G‐1 effective *in vivo* in reducing tumor progression, but protective effects against the formation of new tumors even after secondary challenge using the same cell line were observed, suggesting immune memory protection against rechallenge (Natale et al. 2018). G‐1 is a racemic mixture, and the specific enantiomer responsible for the activity, LNS8801, was identified (Natale and Garyantes 2021). LNS8801 was shown to reduce cell proliferation, cell invasion, and c‐Myc levels. LNS8801 is currently in Phase 1/2 trial (NCT04130516) either alone or with an immune anti‐checkpoint inhibitor, pembrolizumab, and has demonstrated activity in melanoma and other solid tumors (Rodon et al. 2023; Shoushtari 2023). Based on these encouraging results we decided to carry out an *in vivo* study using LNS8801 and our melanoma‐prone transgenic mouse model, TGS.

The TGS model was established by crosses between the original transgenic melanoma mouse model, TG‐3 (Eddy et al. 2023; Zhu et al. 1998), and the hairless model, SKH (Benavides et al. 2009). The benefit of using this model is the spontaneous development of pigmented lesions that are visible without the fur; furthermore, these pigmented lesions can be tracked using a small animal imaging system (IVIS) as described (Eddy et al. 2023).

We initiated the *in vivo* study to investigate the putative therapeutic efficacy of LNS8801 in immunocompetent TGS mice in a longitudinal study of 32 weeks with or without UV irradiation. We only selected heterozygous TGS for the study due to their longevity (Eddy et al. 2023). A total of 48 heterozygous TGS mice at 8 weeks old were divided into the following groups with equal number of male and female mice in each group: (1) No UVB and vehicle (oral gavage, three times a week, 13% DMSO, 82% sesame oil and 5% 200 proof ethanol), (2) UVB (30 mJ/cm^2^) once a week and vehicle, (oral gavage, three times a week, 13% DMSO, 82% sesame oil and 5% 200 proof ethanol), (3) No UVB and LNS8801 (oral gavage, three times a week at 1 mg/kg), and (4) UVB (30 mJ/cm^2^) once a week plus LNS8801 (oral gavage, three times a week at 1 mg/kg). For the two groups not exposed to UVB, IVIS images of the dorsal side of the animal were taken, along with an aliquot of blood and measurement of body weights every 4 weeks. For the two groups exposed to UVB, the images, blood samples, and weight of each mouse were performed every 2 weeks. Whole blood samples taken were centrifuged and the plasma was collected and stored at −80°C for further analyses. At 32 weeks from the start of treatment, the study was terminated, and samples were taken of tumors, nearby normal skin, lymph nodes, brains, lungs, and livers.

**FIGURE 1:**
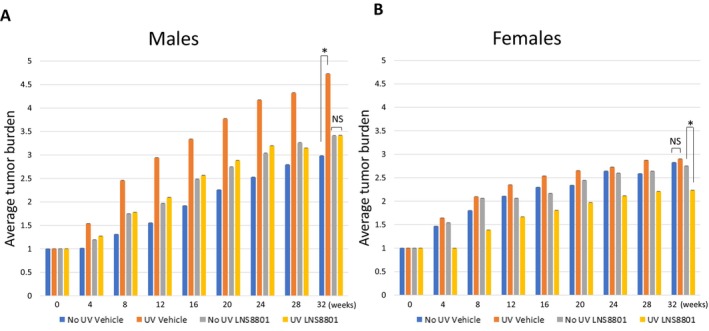
Average tumor burden in mice treated with vehicle or LNS8801. (A) Male and (B) female mice were treated three times a week for 32 weeks with vehicle or LNS8801. UVB exposure was performed once a week at 30 mJ/cm^2^. A small animal imaging system, IVIS, was used to acquire images. Tumor burden was calculated using ImageJ software as described (Eddy et al. 2023). The average tumor burden for each treatment group is normalized to its respective week 0. *p ≤ 0.05, NS stands for no significance. Statistical significance was calculated using a one‐way ANOVA with Bonferroni post hoc analysis.

Calculation of tumor burden of each mouse throughout the study was described earlier (Eddy et al. 2023). A statistically significant increase in tumor burden from as early as 4 weeks after initiation of the experiment was observed in TGS male mice treated with vehicle and UVB (Figure 1A). In contrast, in LNS8801 treated male TGS, the addition of UVB irradiation did not further increase tumor burden (Figure 1A). These results suggest that treatment with LNS8801 protected the male mice from UVB‐induced augmented tumor burden. In contrast, in vehicle‐treated TGS female mice, UVB did not further increase tumor burden as observed in male mice (Figure 1B). Furthermore, the addition of UVB to LNS8801‐treated female mice also exhibited significantly lower tumor burden (Figure 1B), similar to the observation in LNS8801‐treated male mice (Figure 1A). LNS8801 appeared to protect the UVB‐enhanced tumor burden in both male and female mice (Figure 1A,B). Taken together, these results confirmed earlier results with G‐1, the agonist of GPER. It appears that the female sex hormone, estrogen, has a protective role in melanomagenesis induction via UVB, a well‐known environmental toxicant. Female mice have a much higher basal level of estrogen in circulation allowing for increased activation of GPER in melanocytes leading to constant protection from initiation of tumors and tumor progression to melanoma. A second explanation could be that the increased exposure of UVB led to increased mutations leading to Thymine‐Thymine dimers that have been shown to increase p53 activity and upregulate the production of epidermal melanin (Eller, Yaar, and Gilchrest 1994). Both the enhanced activity of the tumor suppressor p53 and amplified melanin production will create a protective shielding environment for the melanocytes not to be transformed by UVB.

At the end of the study after 32 weeks, H&E histopathology was performed with excised liver samples from randomly selected male and female mice from each of the treatment groups. Minimal small macrophage aggregates with vesiculate nuclei were observed in some mice at the 32‐week time point with no other significant microscopic observations in all liver tissue sections, suggesting no noticeable toxicity.

**FIGURE 2:**
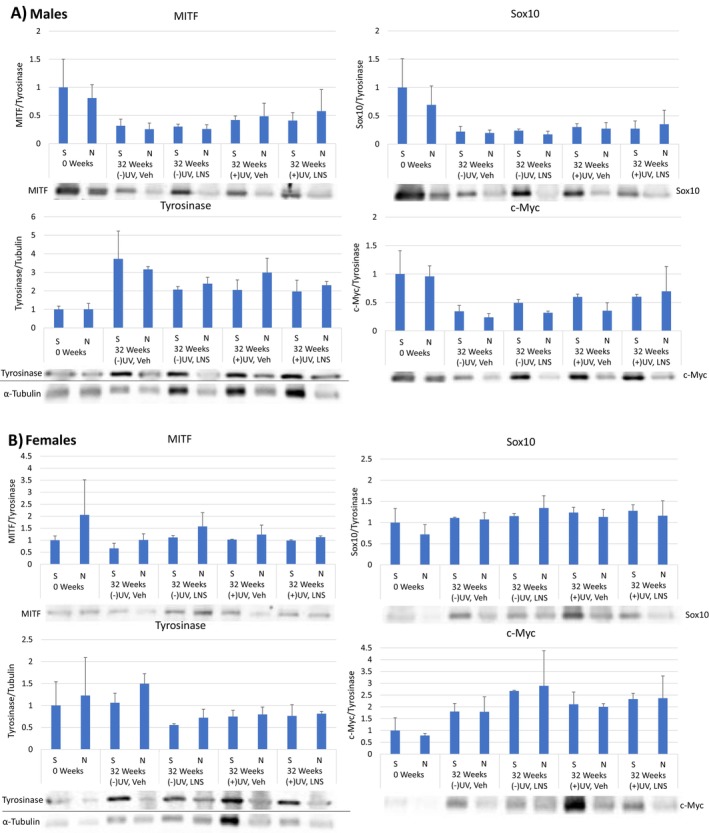
Protein expression levels of MITF, Sox10, Tyrosinase, and c‐Myc in vehicle or LNS8801 treated TGS mice. Representative westerns of (A) male and (B) female mice that were treated with vehicle or LNS8801 for 32 weeks. UVB exposure was once a week at 30 mJ/cm^2^. S = normal skin, N = raised nevi, Veh = vehicle, LNS = LNS8801. At 32 weeks, samples of raised nevi or nearby normal skin without nevi were taken and processed for protein lysate. The protein lysates were then used in western immunoblots and probed for MITF, Sox10, Tyrosinase, and c‐Myc. Protein band intensities were scanned and quantified with ImageJ; average of at least two independent westerns was calculated. MITF, Sox10, and c‐Myc levels were all normalized to Tyrosinase to limit to melanocytes. Tyrosinase was normalized to tubulin. Statistical significance was calculated using a one‐way ANOVA with Bonferroni post hoc analysis; no significance was observed.

A portion of the tumor and adjacent normal skin without any pigmented lesions were collected, respectively, at the termination of the study at 32 weeks and subjected to protein lysate preparation for western immunoblots to probe for Tyrosinase, MITF, Sox10, and c‐Myc. Each of the lanes consisted of 3–4 independent skin or nevi samples collected, each western was performed at least two times, and the values shown are the average of the scans done with Image J. A representative image of each western is shown below its corresponding quantification (Figure 2). Expression of MITF, Sox10, and c‐Myc was normalized to Tyrosinase to limit to melanocytes. For levels of Tyrosinase, we used α‐tubulin for normalization. We normalized the levels of protein expression for skin (without any pigmented lesions) set at 1 and the data are expressed as percent of skin. MITF, a master regulator for melanocytic cell lineage, showed the highest levels at the initiation of the study (designated as week 0); the TGS mice were 8 weeks old at this point. As the mice aged throughout the experiment, no significant alterations in MITF levels were detected regardless of if the mice were given LNS8801 (Figure 2A). Similar observations in Sox10, a transcription factor that participates in terminal differentiation and maintenance of melanocytic phenotypes, were observed (Figure 2A). It is not surprising that as the TGS mice aged, levels of Tyrosinase increased. Tyrosinase is a rate‐limiting enzyme for melanin production; the elevated Tyrosinase levels detected are likely due to the increased melanin production in pigmented lesions (Figure 2A). c‐Myc is a multifunctional transcription factor that has been implicated in various cell proliferation and cell transformation tasks. Earlier, Ridky and co‐workers showed a reduction in c‐Myc protein expression in melanocytes in the presence of LNS8801; our results confirmed the earlier results when comparing Week 32 to Week 0 in male mice (Figure 2A). In female TGS mice, similar results were seen in MITF and Sox10 as in males (Figure 2B). In contrast, Tyrosinase levels did not increase as we observed for male mice (Figure 2B). c‐Myc levels were also higher but not statistically significant in LNS8801‐treated mice with or without UVB (Figure 2B). None of the protein markers yielded statistically significant differences between Week 0 and Week 32, UVB versus no UVB, or LNS8801 versus vehicle. Lack of significant difference may represent a limitation of assessing protein lysates derived from heterogenous tissues, despite the normalization of each protein maker with corresponding Tyrosinase levels.

Interleukin‐10 (IL‐10) and interferon‐γ (IFN‐γ) are immunomodulatory cytokines, and they are normally detected in a subpopulation of T‐helper and T‐regulatory cells. Earlier studies by others demonstrated that simultaneous application of IL‐10 and IFN‐γ significantly inhibited the stimulation of CD4^+^ helper T cells by dendritic cells (Hirata et al. 2009). IL‐10 serves many key functions, one of which is to aid in the development of memory CD8^+^T cells and natural killer cells (Foulds, Rotte, and Seder 2006; Lauw et al. 2000). Activation of GPER was shown earlier to increase IL‐10 levels (Hedrich and Bream 2010). IFN‐γ is secreted by T helper 1(Th1) cells when they are activated by the immune system in response to many stimuli including the detection of cancer cells. The secreted IFN‐γ then aids in B cell differentiation and proliferation (Snapper and Paul 1987). We were interested to know if the observed reduced tumor burden by LNS8801 may be mediated through one or both IL‐10 and IFN‐γ. We took advantage of the IFN‐γ and IL‐10‐ELISA kits designed to measure the level of circulating IFN‐γ or IL‐10 in the collected blood plasma samples across treatment groups throughout the study.

**FIGURE 3:**
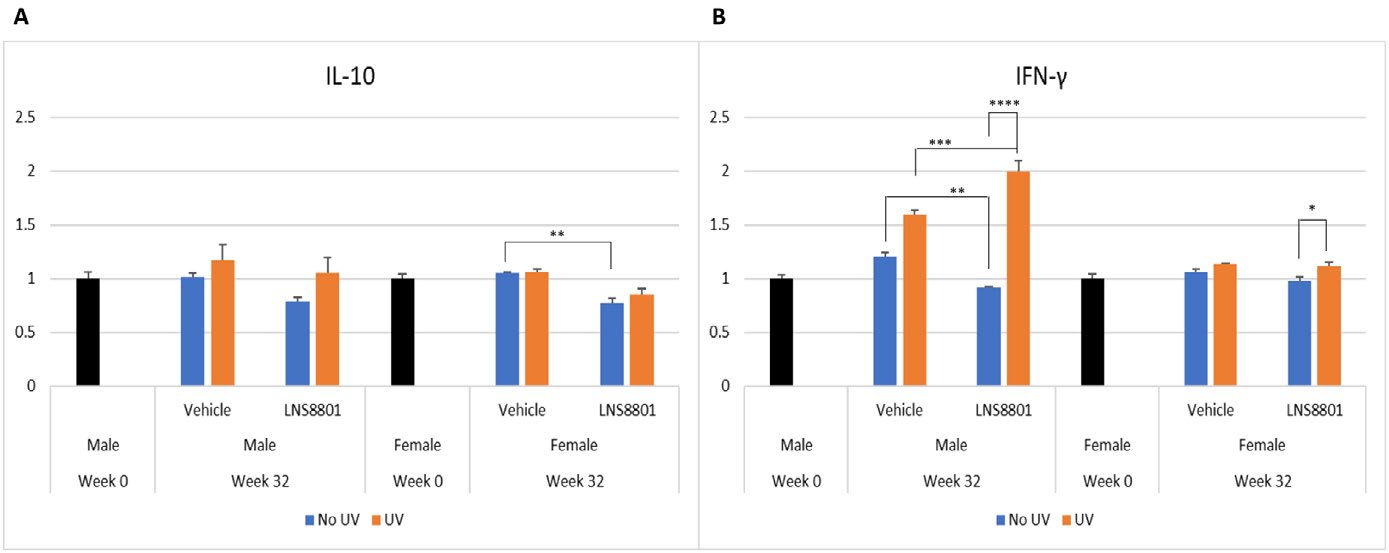
Circulating levels of IL‐10 and IFN‐γ in blood plasma samples from vehicle or LNS8801 treated TGS mice. ELISA kits were used to measure levels of (A) IL‐10 and (B) IFN‐γ in the circulating blood plasma of both male and female mice. Mice were treated with vehicle or LNS8801 for 32 weeks. UVB exposure was once a week at a dosage of 30 mJ/cm^2^. Whole blood was taken via retroorbital bleeding and then centrifuged to isolate the plasma. *p ≤ 0.05, **p ≤ 0.01, ***p ≤ 0.001, ****p ≤ 0.0001. Statistical significance was calculated using a one‐way ANOVA with Bonferroni post hoc analysis.

For IL‐10, the addition of UVB exposure increased IL‐10 levels in TGS male mice regardless of whether the mice were treated with vehicle or LNS8801 but was not statistically significant (Figure 3A). In contrast, TGS female mice displayed a significant decrease in IL‐10 when treated with LNS8801 without UVB (Figure 3A). These results suggest that the protective properties by LNS8801 in tumor burden observed in UVB‐exposed TGS female mice likely were not mediated by IL‐10. For IFN‐γ, elevated IFN‐γ levels were detected in male TGS in the UVB‐irradiated group also when LNS8801was added. In the absence of UVB, a reduction in IFN‐γ was seen in LNS8801 treated male mice (Figure 3B). For TGS female mice, raised IFN‐γ levels were only detected in UVB plus LNS8801‐treated mice (Figure 3B), suggesting that increased IFN‐γ may participate in the protective activity in tumor burden from LNS8801 in the presence of UVB.

## 2 Summary

The current study took advantage of our melanoma‐prone TGS mice with an intact immune system to assess the long‐term therapeutic and/or protective effect of LNS8801, the active enantiomer of G‐1. Blood plasma, tumors, and nearby normal skin samples were taken at various timepoints for analyses in protein markers and two cytokines. Preclinical therapeutic testing is critical and essential in drug development. Many of these studies rely on the *in vivo* results from the gold standards of allograft and/or xenograft studies. These graft studies are short‐term, in some cases are performed in immunocompromised mice, and the consequences are frequent failure in therapeutic responses of patients. TGS mice with an intact immune system exhibit onset and progression of melanoma similar as described for human melanoma patients (Eddy et al. 2023).

Activation of GPER via LNS8801 was shown to decrease cell proliferation and increase cell differentiation in melanocytes, similar to activation of GPER via the hormone estrogen. Previous research has shown that individuals with higher levels of estrogen have a better prognosis when diagnosed with melanoma than those with lower levels (White 1959). It is known that LNS8801 does not activate the classical estrogen receptors thus it could be a viable option for the treatment of melanoma in both males and females without any of the side effects associated with the activation of the classical estrogen receptors. Our results showed that in both male and female TGS mice, treatment with LNS8801 led to a reduction in UVB‐induced increase in tumor burden.

Earlier studies suggest that activation of GPER promotes cell differentiation as evident by the upregulated melanocytic differentiation markers. Our assessment of differentiation markers by western immunoblots of tissue protein lysates did not show statistically significant changes in melanocytic specific markers, MITF and Sox10. c‐Myc, a transcription marker that antagonizes differentiation and promotes cell proliferation and survival, showed contrasting results in females as reported earlier, while in males a reduction in c‐Myc levels was detected. Evaluation of two immunomodulatory cytokines, IL‐10 and IFN‐γ, showed that in TGS male mice UVB did not appear to be the critical player responsible for the IL‐10 levels. However, elevated circulating IFN‐γ levels were detected in both UVB‐exposed and LNS8801‐treated male and female TGS mice. It is possible females may have a more favorable prognosis than males for treatment with LNS8801 since they have higher basal levels of estrogen in their body so they would already have higher GPER activation before treatment with LNS8801. Further experiments will need to be performed to see if any other anti‐tumorigenic and inflammatory cytokines are altered due to treatment with LNS8801, along with examination of other melanocyte differentiation markers to detect any potential protective effect in our mouse model. Additional studies are needed to determine how UVB irradiation and sex are modulating the activities of LNS8801 and what the mechanisms may be.

## Ethics Statement

The animal study protocol was approved by the Institutional Review Board of Rutgers University (protocol number: 0999900047 and approved on 2/8/2022). Data and protocols will be made available upon request.

## Conflicts of Interest

The authors declare no conflicts of interest.

## Data Availability

The data that support the findings of this study are available from the corresponding author upon reasonable request.
